# Brain MRS correlates with mitochondrial dysfunction biomarkers in MELAS‐associated mtDNA mutations

**DOI:** 10.1002/acn3.51329

**Published:** 2021-05-05

**Authors:** Laura L. Gramegna, Stefania Evangelisti, Lidia Di Vito, Chiara La Morgia, Alessandra Maresca, Leonardo Caporali, Giulia Amore, Lia Talozzi, Claudio Bianchini, Claudia Testa, David N. Manners, Irene Cortesi, Maria L. Valentino, Rocco Liguori, Valerio Carelli, Caterina Tonon, Raffaele Lodi

**Affiliations:** ^1^ IRCCS Istituto delle Scienze Neurologiche di Bologna Functional and Molecular Neuroimaging Unit Bologna Italy; ^2^ Department of Biomedical and NeuroMotor Sciences University of Bologna Bologna Italy; ^3^ IRCCS Istituto delle Scienze Neurologiche di Bologna UOC Clinica Neurologica Bologna Italy; ^4^ Department of Physics and Astronomy University of Bologna Bologna Italy

## Abstract

**Objective:**

The purpose of this study was to investigate correlations between brain proton magnetic resonance spectroscopy (^1^H‐MRS) findings with serum biomarkers and heteroplasmy of mitochondrial DNA (mtDNA) mutations. This study enrolled patients carrying mtDNA mutations associated with Mitochondrial Encephalomyopathy, Lactic Acidosis, and Stroke‐like episodes (MELAS), and MELAS‐Spectrum Syndrome (MSS).

**Methods:**

Consecutive patients carrying mtDNA mutations associated with MELAS and MSS were recruited and their serum concentrations of lactate, alanine, and heteroplasmic mtDNA mutant load were evaluated. The brain protocol included single‐voxel ^1^H‐MRS (1.5T) in the medial parieto‐occipital cortex (MPOC), left cerebellar hemisphere, parieto‐occipital white matter (POWM), and lateral ventricles. Relative metabolite concentrations of N‐acetyl‐aspartate (NAA), choline (Cho), and myo‐inositol (mI) were estimated relative to creatine (Cr), using LCModel 6.3.

**Results:**

Six patients with MELAS (age 28 ± 13 years, 3 [50%] female) and 17 with MSS (age 45 ± 11 years, 7 [41%] female) and 39 sex‐ and age‐matched healthy controls (HC) were enrolled. These patients demonstrated a lower NAA/Cr ratio in MPOC compared to HC (*p* = 0.006), which inversely correlated with serum lactate (*p* = 0.021, rho = −0.68) and muscle mtDNA heteroplasmy (*p* < 0.001, rho = −0.80). Similarly, in the cerebellum patients had lower NAA/Cr (*p* < 0.001), Cho/Cr (*p* = 0.002), and NAA/mI (*p* = 0.001) ratios, which negatively correlated with mtDNA blood heteroplasmy (*p* = 0.001, rho = −0.81) and with alanine (*p* = 0.050, rho = −0.67). Ventricular lactate was present in 78.3% (18/23) of patients, correlating with serum lactate (*p* = 0.024, rho = 0.58).

**Conclusion:**

Correlations were found between the peripheral and biochemical markers of mitochondrial dysfunction and brain in vivo markers of neurodegeneration, supporting the use of both biomarkers as signatures of MELAS and MSS disease, to evaluate the efficacy of potential treatments.

## Introduction

MELAS (Mitochondrial Encephalopathy, Lactic Acidosis, and Stroke‐like episodes) syndrome is a rare maternally inherited, multi‐systemic disorder with a generally poor yet variable prognosis.^1^, ^2^, ^3^, ^4^

The most common mutation associated with MELAS is the mitochondrial DNA (mtDNA) m.3243A > G point mutation in the *MT‐TL1* gene encoding for tRNA leucine.^5^

However, there are less frequent mutations (e.g., m.3271T > C and m.3252A > G) in the same gene, but also in the *MT‐TL2* gene (encoding the second tRNA leucine), and rare mutations in other mtDNA genes (e.g., *MT‐ND3‐6* encoding subunits of complex I, *MT‐CO2,* and *MT‐CO3* encoding subunits of complex IV) that have been reported in other patients with MELAS.^6^

Although stroke‐like episodes (SLE) are the hallmark of MELAS syndrome in its strict definition,^4^ these mutations but particularly the commonest, m.3243A > G, may be characterized by a very broad spectrum of clinical manifestations.^7^, ^8^, ^9^, ^10^ This spectrum includes incomplete phenotypes characterized by dementia, epilepsy, lactic acidosis, myopathy, cardiomyopathy, renal failure, gastro‐intestinal dysmotility, retinal dystrophy and maculopathy, recurrent headaches, psychiatric involvement, cerebellar ataxia, hearing impairment, diabetes, and short stature. We here define as MELAS‐spectrum syndrome (MSS)^1^, ^2^, ^3^, ^7^, ^8^, ^9^, ^10^ such clinical manifestations in the absence of SLE. Central nervous system involvement typically indicates a worse prognosis.^11^ In particular, a recent study of a UK cohort of 238 adult m.3243A > G carriers demonstrated that the full‐blown MELAS syndrome occurs in about 17% of patients,^11^, ^12^ whereas a retrospective study from the Nationwide Italian Collaborative Network of Mitochondrial Diseases that characterized 126 m.3243A > G mutation carriers reported SLE in 40.5% of patients.^9^

Patients harboring mtDNA mutations frequently present with mutant and wild‐type mtDNA molecules coexisting within the same cell, a condition defined as heteroplasmy.^7^ The distribution of mutant and wild‐type mtDNA may vary widely among different tissues and among cells within the same tissue, constituting a genetic mosaicism.^13^ Heteroplasmic mutation load is considered a major determinant of the phenotype in mitochondrial diseases although loose genotype–phenotype correlation has been long debated for MELAS.^14^, ^15^ In carriers of the m.3243A > G mutation, it has been demonstrated that both skeletal muscle and blood heteroplasmy levels are correlated with disease severity.^15^

Since oxidative phosphorylation (OXPHOS) is impaired in MELAS patients, pyruvate is converted to lactate, which leads to chronic lactate acidemia. Moreover, a pyruvate‐to‐alanine conversion is also active,^7^ possibly leading to increased serum alanine in MELAS. Serum lactate acidemia is a defining feature in MELAS that reflects clinical severity and possibly the heteroplasmic mutant load, whereas serum alanine has also been recently proposed as a serum marker of OXPHOS dysfunctions.^7^

Conventional brain magnetic resonance imaging (MRI) findings in MELAS syndrome include stroke‐like lesions (SLLs) in regions not corresponding to vascular territories, a peculiar pattern of gyral necrosis affecting the cortex and juxtacortical white matter referred to as the “black toenails sign,” signal changes in pallidal nuclei, nonspecific T_2_ white matter hyperintensity, and supratentorial and infratentorial brain atrophy.^16^, ^17^ The prevalence of all these features in MELAS and MSS patients remains unknown.

Brain proton magnetic resonance spectroscopy (^1^H‐MRS) allows in vivo evaluation of metabolism in specific volumes of interest (VOIs). The reduction of N‐acetyl‐aspartate (NAA) content may be related to neuro‐axonal degeneration^18^ or to mitochondrial dysfunction,^19^, ^20^ both of which are potentially reversible after effective therapeutic interventions.^21^ Increased myo‐inositol (mI) is considered a marker of the glial activation^18^ that occurs in association with neuronal degeneration or loss. Furthermore, brain ^1^H‐MRS studies in MELAS patients have shown the presence of lactate accumulation^8^ as a result of impaired OXPHOS, in common with other mitochondrial diseases,^22^ in both gray and white matter regions. Besides studies in MELAS patients presenting SLEs, there are no previous systematic investigations of ^1^H‐MRS abnormalities in magnetic resonance (MR)‐defined normal‐appearing cortical and white matter VOIs of both MELAS and MSS patients.

This study investigated the correlations between peripheral, molecular, and serum biomarkers with brain in vivo MRS findings in a relatively large cohort of MELAS and MSS patients, with the goal of identifying signatures for brain involvement and contributing to a personalized profile of disease, potentially useful for prognostic and therapeutic purposes.

## Methods

### Subjects

Between 2003 and 2018, consecutive patients with a clinical diagnosis of MELAS and MSS, molecularly confirmed as carrying a mtDNA mutation associated with MELAS, were prospectively referred to the Functional MR Unit from the Mitochondrial Medicine Clinic at the IRCCS Istituto delle Scienze Neurologiche di Bologna, Bellaria Hospital, Bologna (Italy) as part of their diagnostic work‐up. Clinical and biochemical data were retrospectively reviewed from the clinical charts of MELAS and MSS patients routinely followed at the Mitochondrial Medicine Clinic. Their MRI and MRS findings were retrospectively reviewed and processed by the Functional and Molecular Neuroimaging Unit of the IRCCS Istituto delle Scienze Neurologiche di Bologna, Bellaria Hospital, Bologna (Italy). A cohort of healthy controls (HC) was identified to match the sex and age of enrolled patients for each MRI and MRS evaluation. The institutional review board committee approved the study (Comitato Etico Indipendente #1386).

### Clinical and instrumental definition of MELAS and MSS syndrome

Patients were considered to have a MELAS syndrome according to the criteria proposed by Yatsuga et al. 2012^4^ in presence of at least two category A criteria (i.e., headaches with vomiting, seizures, hemiplegia, cortical blindness, acute focal lesions on neuroimaging) and two category B criteria (i.e., high plasma or cerebrospinal fluid lactate, mitochondrial abnormalities on muscle biopsy, MELAS‐related pathogenic mtDNA mutation).

Patients lacking SLEs were defined as having a MSS in the presence of a variable combination of mitochondrial myopathy, hearing impairment, diabetes, lactic acidosis, epilepsy, gastro‐intestinal dysmotility, retinal dystrophy and maculopathy, psychiatric involvement, cerebellar ataxia, short stature, along with a mtDNA mutation associated with MELAS.

### Clinical and instrumental definition of mitochondrial myopathy

Patients were considered to have a myopathy when at least one of the following criteria was satisfied: (1) histological findings of mitochondrial myopathy (i.e. presence of ragged red fibers whether COX+ or COX–, or presence of COX– fibers, or fibers with COX partially depleted at SDH/COX double staining with or without the presence of SDH hyperintense fibers),^23^ (2) electromyographic evidence of myopathic changes, and 3) clinical signs of myopathy at the neurological examination.

### Genetic definition of the MELAS and MSS cohort

DNA was extracted from white blood cells using the Maxwell^®^ 16 blood DNA purification kit (Promega Corporation, Madison, WI, USA) and from skeletal muscle and urinary sediments using a standard phenol‐chloroform protocol. Heteroplasmy of m.3243A > G/MT‐TL1 mutation was quantified by droplet‐digital polymerase chain reaction (dd‐PCR) using PrimePCR Custom Assays (BioRad, Hercules, CA, USA), which discriminated the wildtype from the mutated genomes, and in urine and skeletal muscle using the SNapShot^®^ Multiplex Kit (Thermo Fisher Scientific, Waltham, MA, USA).^15^ The heteroplasmy of other rare mutations (i.e., m.10197G > A/MT‐ND3, m.10191T > C/MT‐ND3, m.3271T > C/MT‐TL1, and m.6597C > A/MT‐CO1) was quantified by restriction fragment length polymorphism (RFLP). Heteroplasmy was expressed as a percentage of mutated mtDNA genomes on total mtDNA (Table 1). Using an online calculator tool developed by Newcastle University, a correction for age was applied for blood and urine levels of m.3243A > G mutation.^24^

**Table 1:** Demographic, genetic, biochemical, neuroimaging, and MRS data of MELAS and MSS patients.

Patient number	Sex	Age (yrs)	Mutation	Myopathy	Myopathy definition			Heteroplasmy1			Serum lactate basal (mg/dl)	Serum alanine (µmol/L )	Age of onset	Polyneuropathy (ENG)	Neurodevelopmental Delay	Migraine	Sensorineural hearing loss	SLE	SLE in the last 3 months	Status Epilepticus	SE in the last 3 months	Epilepsy	Diabetes	CPEO	Comorbidities
					(Histology)	(EMG)	(NE)	Blood	Urine	Muscle															
1	M	26	3243A > G/MT‐TL1	−	−	NA	NA	25%	38%	NA	7,7	NA	19	NA	−	+	+	−		−		−	−	−	A
2	F	33	3243A > G/MT‐TL1	+	+	−	−	43%	65%	71%	13	513	18	−	−	+	+	−		−		+	+	−	C
3	F	40	3243A > G/MT‐TL1	+	−	−	+	51%	70%	73%	13	298	15	−	−	−	+	−		−		−	+	−	A, L
4	M	38	3243A > G/MT‐TL1	+	+	−	−	64%	NA	78%	NA	312	15	+SP	−	−	+	−		−		+	+	−	L
5	F	61	3243A > G/MT‐TL1	+	+	−	+	18%	54%	72%	NA	NA	24	−	−	−	+	−		−		−	+	+	L
6	F	56	3243A > G/MT‐TL1	+	+	−	−	5%	16%	35%	14	425	Ch	−	−	+	−	−		+	−	+	+	−	C, M
7	M	58	3243A > G/MT − TL1	+	+	NA	−	NA	71%	73%	NA	NA	30	NA	−	−	+	−		−		+	−	−	C, H, I, M
8	M	28	3243A > G/MT‐TL1	+	+	NA	−	58%	96%	NA	53,9	522	Ch	NA	+	+	−	+	+	+	−	+	+	−	B
9	M	39	3243A > G/MT‐TL1	−	NA	−	−	52%	81%	NA	14	506	In	NA	−	−	−	−		−		−	−	−	D
10	F	39	3243A > G/MT‐TL1	+	+	+	+	46%	NA	61%	25	635	Ch	NA	+	‐	−	−		−		−	−	−	D
11	F	36	3243A > G/MT‐TL1	+	+	+	+	55%	85%	87%	50	561	23	−	−	−	+	−		−		−	+		B
12	F	46	3243A > G/MT‐TL1	+	+	+	+	66%	81%	47%	29	NA	20	−	−	−	+	−		−		−	+	+	E,C,B, L
13	F	26	10197G > A/MT‐ND3	−	NA	−	−	85%	98%	NA	11	NA	12	−	−	+	−	+	+	+	−	+	−	−	G, M
14	M	24	3243A > G/MT‐TL1	+	+	−	−	13%	80%	78%	29	612	18	−	−	−	+	+	+	+	+	+	−	−	−
15	F	59	10191T > C/MT‐ND3	−	−	−	−	1%	NA	48%	NA	343	43	+SP	−	−	+	+	−	+	−	+	−	−	G, I, M
16	M	53	3243A > G/MT‐TL1	+	+	+	−	5%	NA	46%	14	501	Ch	−	−	−	+	−		−		−	+	−	−
17	M	42	3243A > G/MT‐TL1	+	+	−	−	50%	NA	55%	16	618	Ch	−	−	+	+	−		−		−	+	+	C, L
18	M	50	3243A > G/MT‐TL1	+	+	NA	NA	47%	NA	67%	NA	NA	20	NA	−		+	−					+		B
19	M	30	3271T > C/MT‐TL1	+	−	+	−	36%	92%	83%	NA	344	29	−	−	−	−	+	+	+	+	+	−	−	F, M
20	M	58	3243A > G/MT‐TL1	+	+	+	+	21%	62%	76%	27	514	18	+SP	−	+	+	−		−		−	+	−	L
21	F	40	6597C > A/MT‐CO1	+	+	NA	+	30%	70%	95%	NA	NA	30	NA	−	+	+	+	−	+	−	+	−	−	D
22	M	57	3243A > G/MT‐TL1	+	+	NA	−	45%	50%	NA	12	401	Ch	NA	−	−	+	−		−		−	+	+	A, L
23	M	33	3243A > G/MT‐TL1	+	+	−	−	94%	NA		20	NA	28	−	−	−	+	−		−		−	+	−	B

Comorbidities: A. nephropathy, B. Wolff‐Parkinson White or cardiomyopathy, C. GI dysmotility, D. psychiatric disorders, E. dysautonomia, F. aphasia, G. peripheral neuropathy, H. myoclonus, I. ataxia, L. retinal dystrophy, M. cognitive impairment.

EMG: Electromyography, NE: Neurological Examination, ENG: electroneurography.

Ch: childhood, CPEO: chronic progressive external ophthalmoplegia, In: indeterminate, SE: status epilepticus, SL: stroke‐like, SLE: stroke‐like episodes.

^1^ The m.3243A > G heteroplasmy levels of blood and urine were corrected using an online tool. + SP: (mild) Sensory Polyneuropathy. NA: data not available.

### Analysis of serum lactic acid and alanine levels

Serum lactic acid levels at rest were evaluated in all patients (normal value: 5–22 mg/dL). Serum alanine levels were also assessed. Biochemical markers were drawn in the fasting state.

### Brain MR protocol

**Figure 1:**
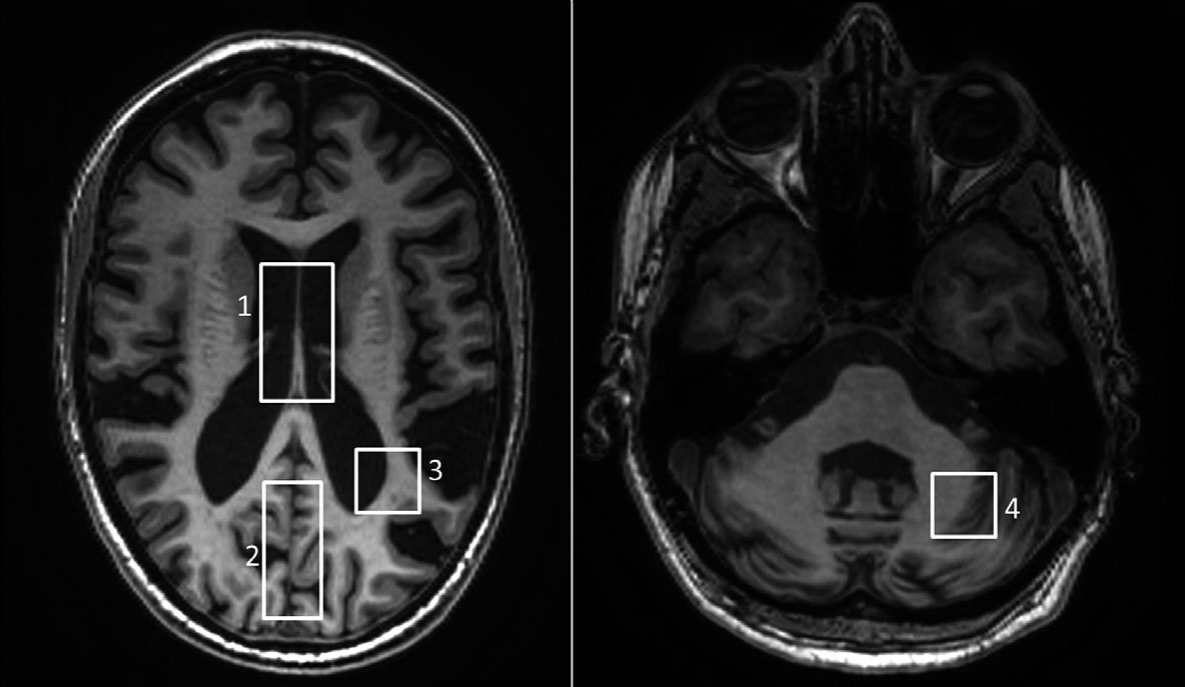
^1^H‐MRS volume of interest localization. (1) Lateral ventricles (TR/TE = 1500/288 msec, volume = 6.4 mL, NEX = 384); (2) Medial parieto‐occipital cortex, MPOC (TR/TE = 4000/35 msec, volume = 18 mL, NEX = 32); (3) Left parieto‐occipital white matter POWM (TR/TE = 4000/35 msec, volume = 8 mL, NEX = 64); (4) Left cerebellar hemisphere (TR/TE = 4000/35 msec, volume = 6 mL, NEX = 64).

Image acquisitions were performed with a 1.5‐T GE Signa HDxt scanner (GE Healthcare, Chicago, IL, USA) equipped with a volumetric head coil. The standardized MR protocol included a volumetric fast spoiled gradient‐echo T1‐weighted image (FSPGR, TR/TE/TI = 12.5/5.1/600 msec, 1 mm^3^ isotropic voxel), an axial fluid‐attenuated inversion recovery image (FLAIR, TR/TE/TI = 8000/84.8/2000 msec, 0.9375 mm in‐plane resolution), and single‐voxel ^1^H‐MR spectroscopy (PRESS) with localizations in: (a) lateral ventricles (TR/TE = 1500/288 msec, volume = 3.7–8.2 mL, NEX = 384); (b) medial parieto‐occipital cortex (MPOC) (TR/TE = 4000/35 msec, volume = 18 mL, NEX = 32); (c) left parieto‐occipital white matter (POWM) (TR/TE = 4000/35 msec, volume = 8 mL, NEX = 64); and (d) left cerebellar hemisphere (TR/TE = 4000/35 msec, volume = 6 mL, NEX = 64) (Fig. 1). The total scan time was 60 min.

### Neuroradiological MRI evaluation

All MRI images were analyzed, in consensus, by two neuroradiologists with 5 and 20 years of experience (LLG and RLo, respectively). They evaluated the presence and morphological characteristics of SLLs, presence of basal ganglia signal alterations, degree of supra‐ and infra‐tentorial atrophy, and degree of white matter hyperintensities.

The degree of supratentorial brain atrophy was evaluated according to validated visual scales.^25^, ^26^ Atrophy of the frontal lobes was evaluated on the coronal slice according to the Davies/Kipps scale,^25^ which considers scores ≥2/4 to be pathological. Atrophy of the parietal/occipital lobes was evaluated in three planes, with scores ≥2/3 considered pathological for this region.^26^ Atrophy of the medial‐temporal lobes was calculated according to Schelten’s scale^26^ and considered pathological in cases scoring ≥2.

To our knowledge, there are no existing validated scales defining atrophy of posterior fossa structures; thus, a pragmatic scale was developed. Vermis atrophy was evaluated in the median‐sagittal T1 FSPGR slice and cerebellar hemispheric atrophy in the coronal T1 FSPGR slices. By comparison with preselected reference images, slight, moderate, and severe atrophy were scored as 1, 2, and 3, respectively (Fig. 1).

White matter hyperintensities were defined as irregular hyperintensities extending into the deep white matter on T2‐weighted scans. Their degree was estimated using a modification Fazekas rating scale of age‐related white matter change,^27^ which scores damage from 0 to 3 as follows: (0) absent; (1) slight: continuous periventricular lines of less than 5 mm and/or non‐confluent subcortical white matter foci of less than 5 mm; (2) moderate: continuous periventricular lines of 5–10 mm and/or non‐confluent subcortical white matter foci of 5 to 10 mm; and (3) severe: more than 10 mm and/or irregular confluent lesions. In a dichotomous classification, a Fazekas score ≥2 was considered severe, whereas a score <2 was considered mild.^28^

### MRS analysis

Spectra were analyzed with LCModel software (version 6.3)^29^ and relative metabolite concentrations were calculated using the creatine (Cr) or mI content as a reference: NAA/Cr, choline (Cho)‐containing compounds Cho/Cr, mI/Cr, and NAA/mI. Lateral ventricles water‐suppressed ^1^H‐MR spectra were pre‐processed with Java‐based Magnetic Resonance User Interface (JMRUI version 3.0) software package based on the Advanced Method for Accurate, Robust, and Efficient Spectral (AMARES) fitting algorithm^30^ with 2 Hz Gaussian filtering followed by –1 Hz exponential filtering. Lac was fitted using the time‐domain semi‐parametric algorithm QUEST, as previously reported.^31^ All MRS spectra were analyzed in consensus by two neuroradiologists (LLG and RLo) who then evaluated for the presence or absence of lactate traces.

### Statistical analysis

Metabolite ratio distributions were tested using the Shapiro–Wilk test. Metabolite concentrations of patients and HCs were compared using the Mann–Whitney test. Correlations of MRS findings with serum lactic acid, alanine, and mtDNA mutation heteroplasmic levels were evaluated with Spearman’s rank correlation. Serum concentrations and mtDNA heteroplasmic levels were also compared between subgroups of patients according to alterations in the Fazekas scale, and supra‐ and infra‐tentorial atrophy using the Mann–Whitney test. Patients and HCs were compared on the Fazekas dichotomous classification with a chi‐square test.

The accuracy of NAA/Cr biomarker to distinguish patients from HCs was further analyzed through receiver operating characteristic (ROC) analysis. The best cut‐off in terms of sensitivity and specificity was identified. All the statistical analyses were conducted within IBM^®^ SPSS^®^ v.25 (IBM, Armonk, NY, USA).

MRS data of this study are available at Mendeley data (http://dx.doi.org/10.17632/3vx6s9twch.1).

## RESULTS

### Patient population

Twenty‐three patients with mtDNA mutations associated with MELAS (ages 42.2 ± 12.1 years; 13 males) and 39 sex‐ and age‐matched HCs were enrolled (Table S1). None of the patients was on idebenone treatment at the time of MRI scan. Demographic, genetic, biochemical, and clinical data of patients are summarized in Table 1. Patients’ neuroimaging and MRS characteristics are summarized in Table 2.

**Table 2:** Neuroradiological and MRS data of and MELAS and MSS patients.

Patient number	Age (yrs)	SLL	Supratentorial atrophy	Infratentorial atrophy	Fazekas scale score > 2	Basal ganglia signal changes	MRS ventricular lactate
1	26	−	−	−	−	−	−
2	33	−	−	−	−	+	−
3	40	−	−	+	+	+	+
4	38	−	+	+	+	+	+
5	61	−	−	+	+	+	+
6	56	−	−	−	−	+	+
7	58	−	−	+	+	+	+
8	28	+	+	+	−	+	+
9	39	−	+	−	−	−	+
10	39	−	+	−	+	−	+
11	36	−	+	+	−	+	+
12	46	−	+	+	−	−	+
13	26	+	−	−	−	−	−
14	24	−	−	−	−	−	−
15	59	+	+	−	−	−	+
16	53	−	−	−	+	+	+
17	42	−	−	−	−	+	+
18	50	−	+	+	−	+	+
19	30	+	−	−	−	−	+
20	58	−	+	+	−	+	+
21	40	+	−	−	−	−	+
22	57	+	+	+	−	+	−
23	33	−	+	−	−	−	+

SLL, stroke‐like lesion.

Six patients (6/23, 26%, ages 28 ± 13 years, 3 [50%] female) presented with full‐blown MELAS syndrome and had at least one SLE in their history (in 4/6 of cases [66.7%] the SLE was associated with status epilepticus, and in 4/6 [66.7%] occurred 3 months before MRS evaluation). Of these patients, 6/6 (100%) had epilepsy, 4/6 (66.7%) had a mitochondrial myopathy, 3/6 (50%) had migraine, 3/6 (50%) had sensorineural hearing loss and 1/6 (17%) had diabetes. Among MELAS patients, 2/6 (33.3%) harbored the m.3243A > G/MT‐TL1 mutation and 4/6 (66.7%) harbored other rare mtDNA mutations: two had the 10197G > A and 10191T > C MT‐ND3 mutation, one had the 3271T > C/MT‐TL1 mutation, and one had the 6597C > A/MT‐CO1 mutation.

Seventeen patients (17/23, 74%, ages 45 ± 11 years, 7 [41%] female) presented MSS syndrome and their clinical picture included epilepsy in 4/17 (24%), mitochondrial myopathy in 15/17 (88%), migraine in 5/17 (29%), sensorineural hearing loss in 14/17 (82%), diabetes in 13/17 (56%). All of them presented the m.3243A > G/MT‐TL1 mutation.

Data in the 39 HCs are shown in Table S1.

### Analysis of serum lactic acid and alanine levels

The average baseline lactic acid level was 31 ± 22 mg/dL for MELAS patients and 20 ± 11 mg/dL for MSS patients (normal values 5–22 mg/dL). The average baseline alanine level was 455 ± 134 µmol/L for MELAS and 480 ± 111 µmol/L for MSS patients (normal values 286–416 µmol/L) (Table 1). Data for serum alanine or lactate were missing in 4 MELAS and 7 MSS patients.

### Brain MRI

**Figure 2:**
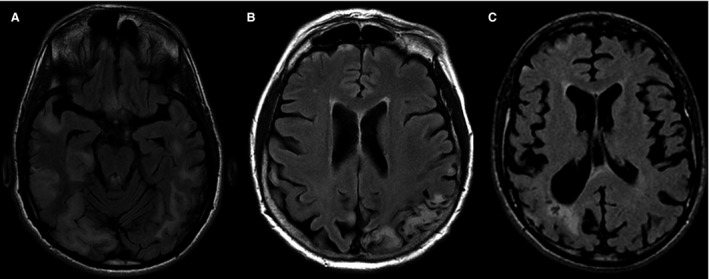
Conventional neuroimaging of three representative MELAS patients. (A) A 28‐year‐old patient harboring the 3243A > G/MT‐TLA1 mutation and presenting the typical features of stroke‐like lesions (SLL). Axial FLAIR T2‐w shows cortical‐subcortical lesions prevalently in the temporo‐parieto‐occipital regions. (B) A 59‐year‐old female with T10191C ND3 mutation (#15) was asymptomatic at MRI scan and had the last stroke‐like episode (SLE) more than 3 months previously. She presented occipital epilepsy from childhood with several episodes of status epilepticus. Axial FLAIR T2‐w showed gyral necrosis affecting the cortex and juxta cortical parieto‐occipital white matter with partial gyral signal suppression on T2/FLAIR sequences, a feature referred to as “black toenails” sign. (C) A 30‐year‐old male with T3271CmtDNA mutation (#19) was asymptomatic at scan evaluation. One month before the scan, he had an SLE that clinically presented with epileptic seizures. Axial FLAIR shows a chronic lesion with focal brain volume reduction in the right parieto‐occipital white matter, characterized by T2 FLAIR hyperintensity with secondary ventricular dilatation and foci of cavitary white matter necrosis.

All subjects completed the whole conventional brain MR protocol. Among MELAS patients (Table 2), 5/6 (83.3%) presented SLLs in the chronic stage. Only one patient (#13), carrying the 10197G > A/MT‐ND3 mutation, was scanned during the acute phase of an SLE that clinically presented with epileptic seizures, which started 5 days before the scan. The brain MRI revealed bilateral, symmetric, T2 hyperintense lesions, affecting predominantly the medial temporal lobe, characterized by prevalent cortical cytotoxic edema. Patient #15, who harbored the 10191G > A/MT‐ND3 mutation, showed gyral necrosis affecting the cortex and juxtacortical POWM, with partial gyral signal suppression on T2/FLAIR sequences, a feature referred to as “black toenails” sign (Fig. 2). Patients #8, #19, #21, and #22 presented chronic T2/FLAIR SLL in areas not corresponding to vascular regions. Patient #21 was previously described in a case report.^32^ Despite patient #8 and #19 presented symptoms in the last 3 months, no increased signal on diffusion‐weighted imaging (DWI) and/or enlargement of previously documented lesions was detected at MRI, so all of them were considered chronic lesions. Patient #14 had a normal scan evaluation, despite having had an SLE 3 months prior.

In the patient cohort, 26.0% (6/23) of patients presented unspecific T2 white matter hyperintensity (i.e., rating scale score of white matter lesions > 2). Even though the percentage of patients with severe Fazekas score was higher in patients than in HC (26.1% [6/23] and 5.9% [1/17], respectively), the difference between the two groups was not statistically significant (*p* = 0.10). More than half of the patients (56.5%, 13/23) had basal ganglia signal changes. One of these patients underwent a brain computed tomography (CT) scan, which confirmed spontaneous hyper‐density related to calcifications. Supratentorial atrophy was present in 47.8% (11/23) of patients for whom a score of ≥2 was obtained in at least one of the evaluated regions. Infratentorial atrophy in the vermis and cerebellar hemisphere was present in 43.5% (10/23) of patients for whom a score ≥2 was obtained in the infratentorial compartment. In patients with MELAS and SLL (*n* = 6), 66.7% (4/6) underwent ^1^H‐MRS examination.

### Brain ^1^H‐MRS

Eleven patients were able to complete the whole MR protocol including all four MRS spectra acquisition; in seven patients three MRS spectra were acquired, in three patients two MRS spectra were acquired, and in two patients only one MRS spectrum was acquired.

In vivo pathological accumulation of lactate in the lateral ventricles was detected in 78.2% (18/23) of patients, which correlated with serum lactate concentration (Table 2). Traces of lactate accumulation were also visually observed in the other MRS localizations: MPOC gray matter in 6/18 (33%) patients, POWM in 9/22 (41%), and cerebellum in 2/11 (18%) patients. Within MPOC, patients had lower NAA/Cr (*p* = 0.006) and NAA/mI (*p* = 0.008) compared to the HCs (Table 3). In the POWM, patients showed lower NAA/Cr (*p* = 0.007), lower NAA/mI (*p* < 0.001), lower Cho/Cr (*p* = 0.005), and higher mI/Cr (*p* = 0.026) than the HCs (Table 3). In the cerebellum, the MELAS and MSS cohort had lower NAA/Cr (*p* < 0.001), NAA/mI (*p* = 0.001), and Cho/Cr (*p* = 0.002) (Table 3). Group comparisons were also conducted excluding MELAS patients and similar results were obtained (Table S2).

**Table 3:** Brain ^1^H‐MRS results: comparisons between metabolite ratios in patient and healthy control groups and correlations between MRS‐derived and serum indicators.

	Patients ratio	HC ratio	*p*‐value		*p*‐value	Rho
Medial parieto‐occipital cortext						
NAA/Cr	1.24 (±0.16)	1.39 (±0.11)	**0.006**	Basal serum lactic acid concentration	**0.021**	−0.68
				Muscle heteroplasmy	**<0.001**	−0.80
Cho/Cr	0.16 (±0.02)	0.17 (±0.02)	0.163			
mI/Cr	0.81 (±0.27)	0.73 (±0.07)	0.667			
NAA/mI	1.63 (±0.40)	1.91 (±0.23)	**0.008**			
Left parieto‐occipital white matter						
NAA/Cr	1.62 (±0.23)	1.80 (±0.14)	**0.007**	Urine heteroplasmy	**0.036**	−0.56
Cho/Cr	0.33 (±0.06)	0.37 (±0.04)	**0.005**			
mI/Cr	1.13 (±0.33)	0.91 (±0.19)	**0.026**			
NAA/mI	1.56 (±0.53)	2.07 (±0.50)	**<0.001**			
Left cerebellar hemisphere						
NAA/Cr	0.96 (±0.15)	1.29 (±0.20)	**<0.001**			
Cho/Cr	0.24 (±0.03)	0.29 (±0.03)	**0.002**			
mI/Cr	0.72 (±0.13)	0.70 (±0.16)	0.862			
NAA/mI	1.37 (±0.29)	1.90 (±0.37)	**0.001**	Alanine serum concentration	**0.050**	−0.67
				Blood heteroplasmy	**0.001**	−0.81

*p*‐values are shown in bold if significant at *p* ≤ 0.05, in underlined if significant after Bonferroni correction. Cho: choline‐containing compounds, Cr: creatine, Patients ratio: MELAS and MELAS‐spectrum syndrome metabolite ratio reported as mean (±standard deviation), HC ratio: healthy control metabolite ratio reported as mean (±standard deviation), mI: myo‐inositol, NAA: N‐acetyl‐aspartate.

ROC analysis revealed that among metabolite ratios, NAA/Cr from the cerebellar localization had the highest accuracy (defined as the area under the curve, AUC) in discriminating patients from HCs (AUC = 94.4% for a threshold value of 1.064: sensitivity = 83.3%, specificity 91.7%). However, good accuracy was also detected for NAA/Cr within MPOC (AUC = 77.5%; for a threshold value of 1.306: sensitivity = 70.6%, specificity 82.4%) and NAA/Cr within POWM (AUC = 74.4%; for a threshold value of 1.799: sensitivity = 85.7%, specificity 57.1%) (Fig. S2).

### Correlations between biochemical, genetic, and in vivo biomarkers

Within MPOC, NAA/Cr correlated negatively with basal serum lactic acid concentration (*p* = 0.021, rho = −0.68) and with muscle mtDNA heteroplasmy (*p* = <0.001, rho = −0.80). In the POWM, NAA/Cr correlated negatively with urine mtDNA heteroplasmy (*p* = 0.036, rho = −0.56). Cerebellar NAA/mI correlated negatively with alanine (*p* = 0.050, rho = −0.67) and with mtDNA blood heteroplasmy (*p* = 0.001, rho = −0.81).

## Discussion

This study combined targeted clinical, biochemical, and genetic markers with in vivo brain MRS metabolic biomarkers in a large cohort of patients with mtDNA mutations associated with MELAS and disclosed widespread neurometabolic alterations. Such alterations included reduced NAA content and lactic acid accumulation in MR‐defined regions, namely the cerebral cortex, white matter, and cerebellum. Notably, a significant correlation was evident between the reduction of the neuronal marker NAA and increases in serum lactate or alanine levels, and with heteroplasmic load of mutant mtDNA in blood, urine, and muscle. These findings suggest that NAA reduction in the central nervous system is well correlated with the peripheral mitochondrial dysfunction occurring in patients with mtDNA mutations associated with MELAS, as evaluated by accumulation of alanine and lactic acid, which is mostly contributed by muscle metabolism. Finally, this study showed that the reduction in NAA in the cerebellum had the highest accuracy in distinguishing patients from HCs.

Previous studies have identified patterns of correlation between heteroplasmic load of mutant mtDNA and disease burden or progression in patients with the m.3243A > G mutations. Skeletal muscle m.3243A > G heteroplasmy was strongly associated with disease burden, as assessed by the Newcastle Mitochondrial Disease Adult Scale (NMDAS). Additionally, age‐adjusted blood loads of m.3243A > G heteroplasmy were strongly associated with disease progression as assessed by NMDAS over time (median follow‐up time was 3.75 years).^15^ In patients with mtDNA mutations associated with MELAS it has been argued that the relationship between heteroplasmy load and clinical phenotype is not straightforward. However, our results broaden previous findings and corroborate the hypothesis that both skeletal muscle and age‐adjusted blood load of m.3243A > G heteroplasmy may reflect brain pathology in these patients, and thus can be used as a measure to monitor disease severity and progression.

We evaluated the brain biochemical abnormalities in the medial parieto‐occipital cortex, MPOC, and the parieto‐occipital white matter, POWM, as it is established that parieto‐occipital regions are more vulnerable in MELAS.^33^ We detected a significant reduction of NAA/Cr and NAA/mI in both regions. Additionally, we found that neuronal NAA content reduction in the MPOC was significantly correlated with muscle tissue mtDNA heteroplasmy and lactic acidemia. This finding is in line with the notion that neuronal and muscle tissues are post‐mitotic systems sensitive to the presence of mtDNA mutations due to their high‐energy requirements and non‐proliferative status.^15^

A significant reduction in the Cho/Cr and increase in the mI/Cr ratio was detected only in the POWM, which may be related to the combination of reduced myelin content (i.e., reduced Cho/Cr) with the diffuse fibrillary gliosis (i.e., increased mI/Cr) observed in neuropathological studies of brain white matter of patients with MELAS.^34^ In our sample, six patients presented SLLs at MRI evaluation and four of these patients underwent ^1^H‐MRS examination. The metabolite defect (Table S2) remained unchanged if these patients were eliminated from the sample, confirming that the ^1^H‐MRS alterations detected are not secondary to the appearance of SLLs.

Cerebellar metabolism was also evaluated in this study, since it has been established that cerebellar atrophy is a major neuroimaging marker of mitochondrial disorders^35^ due to the degeneration of Purkinje and granular cells.^34^ We confirmed a pattern of severe cerebellar metabolic alteration, as demonstrated by the reduced NAA/Cr and Cho/Cr and increased NAA/mI shown in the cerebellar cortex. ROC analysis showed that the NAA/Cr ratio in the cerebellum was able to distinguish between patients with mtDNA mutations associated with MELAS and controls with high accuracy, sensitivity, and specificity (94.4%, 83.3%, and 91.7%, respectively). Moreover, we observed a strong negative correlation between the age‐adjusted blood levels of m.3243A > G heteroplasmy and reduced NAA/mI, which are markers of neurodegeneration in the early phase of the disease. This is interesting since the degree of age‐adjusted blood levels of m.3243A > G heteroplasmy has been demonstrated to correlate with disease progression.^15^ Altogether, these findings suggest that ^1^H‐MRS may be a supporting investigation in the diagnostic work‐up of suspected cases of MELAS and MSS, especially in the absence of a history of SLEs and that the degree of metabolic dysfunction in the cerebellar hemisphere may also be considered a surrogate marker of progression of the disease, although this needs to be confirmed in follow‐up studies.

To our knowledge, this is the first study that examined MRS alterations in MR‐defined normal‐appearing cortical and white matter regions of interest in the inter‐ictal phase in a large cohort of patients. Previous studies have reported a reduction of NAA or NAA/Cr ratios in different brain regions in small case series,^36^ though these were without comparisons with HCs,^20^, ^37^, ^38^ or in the acute phase of SLE.^39^, ^40^

We did not observe differences in serum metabolites when excluding patients with full‐blown MELAS syndrome from our cohort. This may be related to the low prevalence of MELAS in our case series and to the fact that only one case was scanned in the acute phase of the SLE. Moreover, we may speculate that the SLEs occur at least partly because of intercurrent triggering events specific to each patient. Five patients presented with chronic lesions in areas not corresponding to vascular territories, referring to SLLs, one of them with subcortical cavitary evolution of the lesions, confirming the great heterogeneity in brain MRI features associated with MELAS.^16^ Larger multicenter and longitudinal studies are needed to refine the phenotype and evolution of SLLs in patients with common or rare mutations, and to establish whether the cavitary evolution is related to the different MELAS mutations, the degree of biochemical impairment or the heteroplasmic load. Furthermore, our MRS data confirmed the presence of pathological accumulation of lactate^41^ in the lateral ventricles and the correlation with serum lactate levels. The presence of pathological traces of lactate was also detected in other brain regions such as the cerebral cortex, white matter, and cerebellar hemisphere. Overall, these findings provide in vivo evidence of diffuse and chronic OXPHOS dysfunction in different brain areas in absence of SLEs in patients with either MELAS or MSS.

MELAS is considered one of the possible causes of genetic small vessel disease^42^ even though the exact prevalence and clinical significance of this observation is unknown. In our case series, over a quarter of patients presented white matter changes. This MRI pattern has similar characteristics to T_2_ hyperintensities of probable vascular origin,^43^a finding that may relate to the microvascular endothelial dysfunction observed in patients. However, serum metabolites and heteroplasmy loads did not differ in patients with and without signs of small vessel disease.

Brain atrophy is a recognized late feature of MELAS.^16^ In our series of patients, supratentorial atrophy was observed in nearly half of the cases (48%), and infratentorial atrophy in the cerebellar hemisphere and vermis also occurred in a large proportion (43%) of our patient sample.

In conclusion, the key finding of this study is the correlation of *ex vivo* peripheral and biochemical indicators hallmarking mitochondrial dysfunction (e.g., mtDNA mutation load, lactate acidemia) with in vivo markers of neurodegeneration (i.e., in vivo brain H^1^‐MRS). This correlation provides a rationale for using both biomarkers as surrogate indicators of central nervous system involvement in the disease of patients with MELAS and MSS, for accurate evaluation of natural history at follow‐up and to assess the efficacy of potential treatments.

Overall, these results contribute to patient‐specific genetic and molecular profiling, according to the rapidly evolving field of precision medicine,^44^ ultimately aimed at personalized treatment. Although previous studies have provided preliminary results regarding potential pharmacological targets, such as arginine administration^45^, ^46^ and supplementation of high‐dose taurine for reducing the recurrence of SLEs,^47^ effective therapies have not yet been identified. A major issue for designing pharmacological trials is a stratification of patients into homogenous disease stages, while biochemical and genetic markers could be employed for such a stratification, as well as to monitor disease severity and progression.

The conclusions of this study are limited in part by the small sample size, which may have contributed to the uncertain correlation between alanine serum concentration and cerebellar NAA/mI reduction, as well as the apparent lack of correlation between serum biomarkers and ^1^H‐MRS metabolites in the left parieto‐occipital‐white matter.

## Summary

Our study identified a common diffuse pattern of brain biochemical alterations detected in vivo by ^1^H‐MRS, which correlated with serum biomarkers of mitochondrial dysfunction in patients with mtDNA mutations associated with MELAS. We propose the use of in vivo MRS‐detectable brain metabolites and peripheral serum changes as surrogate markers to determine disease stage and progression. This information may be used to assess the efficacy of future therapeutic trials.

## CONFLICT OF INTEREST

La Morgia reports speaker honoraria from Santhera Pharmaceuticals, from Regulatory Pharmanet, and travel reimbursement from GenSight Biologics, outside the submitted work. Carelli reports travel reimbursement from Santhera Pharmaceuticals, from Stealth BioTherapeutics and from GenSight Biologics, grants from Stealth BioTherapeutics, outside the submitted work. Other authors have nothing to disclose.

## AUTHORS’ CONTRIBUTIONS

LLG, SE, LDV, and CLM drafted the manuscript and analyzed the data. AM, LC, GA, LT, CB, CTe, DNM, MLV, RLi, and IC contributed to the acquisition, analysis, and interpretation of data. VC, CTo, and RLo supervised the study and contributed to the study conception and design. All authors discussed the results and contributed to the final version of the manuscript.

## Supporting information


**Figure S1**. Empirical scale developed to describe the atrophy of posterior fossa structures. Vermis atrophy was evaluated in the sagittal T1 FSPGR slice, and hemispheric atrophy in coronal T1 FSPGR. Slight atrophy was scored as 1, moderate atrophy as 2, and severe atrophy as 3 by comparison with preselected reference images.
**Figure S2**. ROC analysis for NAA/Cr in cerebellar hemisphere, medial parieto‐occipital cortex (MPOC), and left parieto‐occipital white matter (POWM). AUC: area under the curve.
**Table S1**. Demographic data for MELAS and MSS patient and healthy control subgroups are reported for each ^1^H‐MRS localization.
**Table S2**. Comparison of ^1^H‐MRS metabolite ratios for MSS patients compared to healthy controls.
