# Supplementary material for: Brain MRS correlates with mitochondrial dysfunction biomarkers in MELAS‐associated mtDNA mutations
Source: Ann Clin Transl Neurol. 2021 May 5;8(6):1200–11. doi: 10.1002/acn3.51329 (PMC8164862; doi:10.1002/acn3.51329)
Supplement: Supplementary file 1 — Figure S1. Empirical scale developed to describe the atrophy of posterior fossa structures. Vermis atrophy was evaluated in the sagittal T1 FSPGR slice, and hemispheric atrophy in coronal T1 FSPGR. Slight atrophy was scored as 1, moderate atrophy as 2, and severe atrophy as 3 by comparison with preselected reference images. Figure S2. ROC analysis for NAA/Cr in cerebellar hemisphere, medial parieto‐occipital cortex (MPOC), and left parieto‐occipital white matter (POWM). AUC: area under the curve. Table S1. Demographic data for MELAS and MSS patient and healthy control subgroups are reported for each 1H‐MRS localization. Table S2. Comparison of 1H‐MRS metabolite ratios for MSS patients compared to healthy controls. [file ACN3-8-1200-s001.docx]

**Supplementary Materials**

**Supplementary Figure 1.** Empirical scale developed to describe the atrophy of posterior fossa structures. Vermis atrophy was evaluated in the sagittal T1 FSPGR slice, and hemispheric atrophy in coronal T1 FSPGR. Slight atrophy was scored as 1, moderate atrophy as 2 and severe atrophy as 3 by comparison with preselected reference images.





**Supplementary Figure 2.** ROC analysis for NAA/Cr in cerebellar hemisphere, medial parieto-occipital cortex (MPOC) and left parieto-occipital white matter (POWM). AUC: area under the curve

**
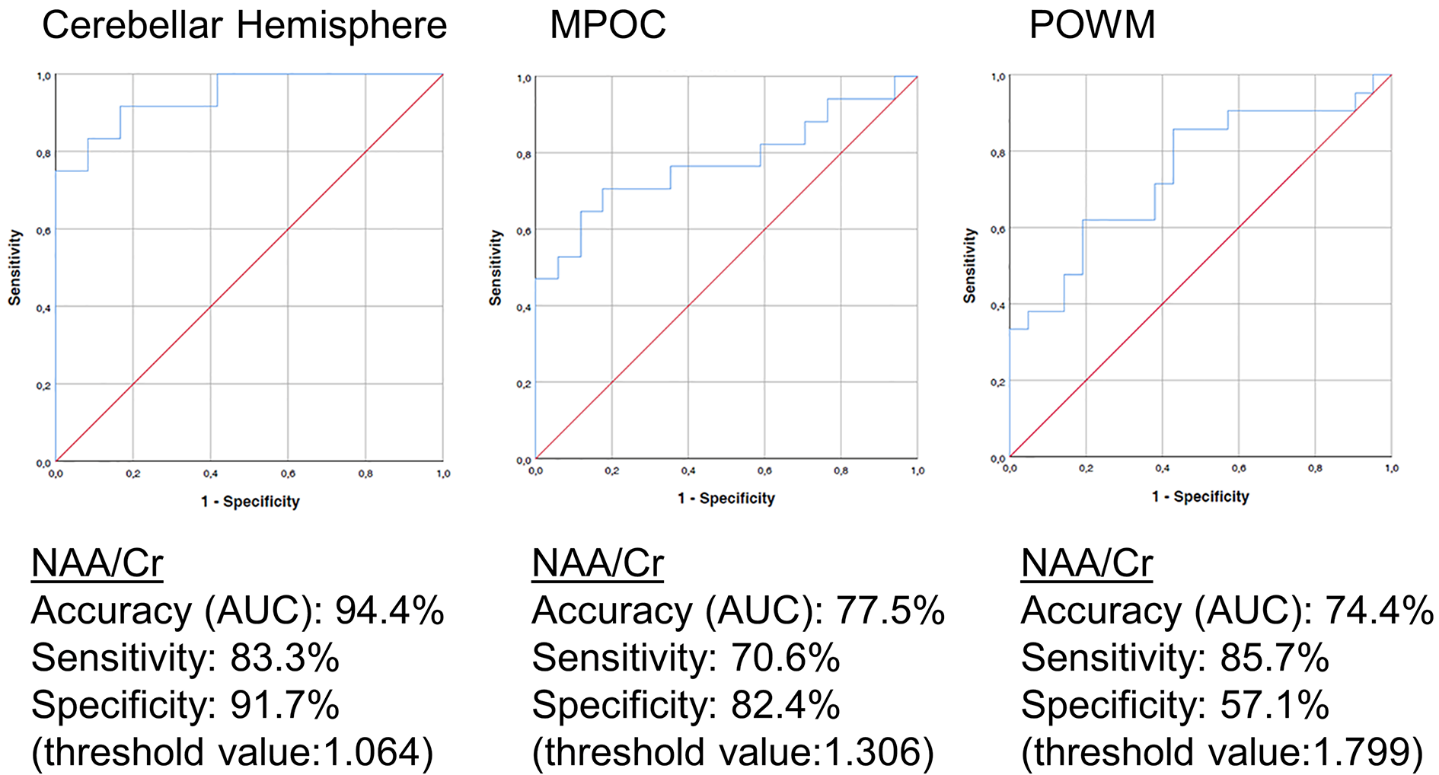
**

**Supplementary Table 1.** Demographic data for MELAS and MSS patient and healthy control subgroups are reported for each ^1^H-MRS localization.

|  | ***MELAS and MSS patients*** | ***Healthy Controls*** | ***MSS patients*** | ***Healthy Controls*** |
| --- | --- | --- | --- | --- |
|  | *MPOC* | | | |
| *N (M/F)* | *17 (9/8)* | *17 (9/8)* | 14 (8/6) | 14 (8/6) |
| Age (years) | 43.2 ± 12.4 | 43.8 ± 11.6 | 42.9 ± 12.1 | 43.8 ± 11.4 |
|  | *POWM* | | | |
| *N (M/F)* | 21 (12/9) | 21 (12/9) | 17 (10/7) | 17 (10/7) |
| Age (years) | 42.5 ± 11.4 | 42.1 ± 13.0 | 43.1 ± 11.1 | 42.4 ± 12.9 |
|  | *Cerebellar hemisphere* | | | |
| *N (M/F)* | 12 (5/7) | 12 (5/7) | 10 (4/6) | 10 (4/6) |
| Age (years) | 44.0 ± 13.3 | 43.4 ± 14.4 | 43.4 ± 12.5 | 42.8 ± 13.4 |

MELAS: Mitochondrial Encephalomyopathy, Lactic Acidosis, and Stroke-like episodes, SLL: Stroke like lesion, M: Male, F: Female, MSS: Melas-Spectrum Syndrome

**Supplementary Table 2.** Comparison of ^1^H-MRS metabolite ratios for MSS patients compared to healthy controls.

| ***Metabolite ratio*** | ***MSS Patients*** | ***Healthy Controls*** | ***p-value*** |
| --- | --- | --- | --- |
| *MPOC* | | | |
| NAA/Cr | 1.26(±0.14) | 1.41(±0.11) | **0.008** |
| Cho/Cr | 0.16(±0.01) | 0.17(±0.02) | 0.089 |
| mI/Cr | 0.72(±0.08) | 0.74(±0.07) | 0.646 |
| NAA/mI | 1.76(±0.28) | 1.93(±0.25) | 0.054 |
| *POWM* | | | |
| NAA/Cr | 1.65(±0.20) | 1.81(±0.12) | **0.013** |
| Cho/Cr | 0.32(±0.05) | 0.38(±0.04) | **0.006** |
| mI/Cr | 1.05(±0.27) | 0.91(±0.20) | 0.221 |
| NAA/mI | 1.68(±0.50) | 2.10(±0.54) | **0.004** |
| *Cerebellar hemisphere* | | | |
| NAA/Cr | 0.96(±0.16) | 1.24(±0.15) | **0.001** |
| Cho/Cr | 0.24(±0.03) | 0.29(±0.03) | **0.009** |
| mI/Cr | 0.75(±0.11) | 0.68(±0.17) | 0.406 |
| NAA/mI | 1.30(±0.23) | 1.87(±0.37) | **0.001** |

MSS patients and healthy control metabolite ratio reported as mean (±standard deviation). P-values of Mann-Whitney tests are shown in bold if significant (p<0.05), in bold if significant after Bonferroni correction (p<0.0125). MSS: Melas-Spectrum Syndrome, MELAS: Mitochondrial Encephalomyopathy, Lactic Acidosis, and Stroke-like episodes, MPOC: medial parieto-occipital cortex, POWM: parieto-occipital white matter, Cr: creatine, mI: myo-inositol (mI), NAA: N-acetyl-aspartate, Cho: choline-containing compounds.
